# An autopsy study of combined pulmonary fibrosis and emphysema: correlations among clinical, radiological, and pathological features

**DOI:** 10.1186/1471-2466-14-104

**Published:** 2014-06-28

**Authors:** Minoru Inomata, Soichiro Ikushima, Nobuyasu Awano, Keisuke Kondoh, Kohta Satake, Masahiro Masuo, Yuji Kusunoki, Atsuko Moriya, Hiroyuki Kamiya, Tsunehiro Ando, Noriyo Yanagawa, Toshio Kumasaka, Takashi Ogura, Fumikazu Sakai, Arata Azuma, Akihiko Gemma, Tamiko Takemura

**Affiliations:** 1 Department of Respiratory Medicine, Japanese Red Cross Medical Centre, 4-1-22 Hiroo, Shibuyaku, Tokyo 150-8953, Japan; 2 Department of Pulmonary Medicine and Oncology, Graduate School of Medicine, Nippon Medical School, 1-1-5 Sendagi, Bunkyoku, Tokyo 113-8603, Japan; 3 Department of Infectious Diseases, Japanese Red Cross Medical Centre, 4-1-22 Hiroo, Shibuyaku, Tokyo 150-8953, Japan; 4 Department of Radiology, Tokyo Metropolitan Cancer and Infectious Diseases Centre, Komagome Hospital, 18-22, Honkomagome 3chome, Bunkyo-ku, Tokyo 113-8677, Japan; 5 Department of Pathology, Japanese Red Cross Medical Centre, 4-1-22 Hiroo, Shibuyaku, Tokyo 150-8953, Japan; 6 Department of Respiratory Medicine, Kanagawa Cardiovascular and Respiratory Centre, 6-16-1 Tomioka-higashi, Kanazawa-ku, Yokohama, Kanagawa 236-0051, Japan; 7 Department of Diagnostic Radiology, Saitama International Medical Centre, Saitama Medical University, 1397-1 Yamane, Hidaka City, Saitama 350-1298, Japan

**Keywords:** Thick-walled cystic lesion, Combined pulmonary fibrosis and emphysema, Autopsy, Idiopathic pulmonary fibrosis, Emphysema, Fibroblastic foci

## Abstract

**Background:**

Clinical evaluation to differentiate the characteristic features of pulmonary fibrosis and emphysema is often difficult in patients with combined pulmonary fibrosis and emphysema (CPFE), but diagnosis of pulmonary fibrosis is important for evaluating treatment options and the risk of acute exacerbation of interstitial pneumonia of such patients. As far as we know, it is the first report describing a correlation among clinical, radiological, and whole-lung pathological features in an autopsy cases of CPFE patients.

**Methods:**

Experts retrospectively reviewed the clinical charts and examined chest computed tomography (CT) images and pathological findings of an autopsy series of 22 CPFE patients, and compared these with findings from 8 idiopathic pulmonary fibrosis (IPF) patients and 17 emphysema-alone patients.

**Results:**

All patients had a history of heavy smoking. Forced expiratory volume in 1 s/forced vital capacity (FEV1/FVC%) was significantly lower in the emphysema-alone group than the CPFE and IPF-alone groups. The percent predicted diffusing capacity of the lung for carbon monoxide (DLCO%) was significantly lower in the CPFE group than the IPF- and emphysema-alone groups. Usual interstitial pneumonia (UIP) pattern was observed radiologically in 15 (68.2%) CPFE and 8 (100%) IPF-alone patients and was pathologically observed in all patients from both groups. Pathologically thick-cystic lesions involving one or more acini with dense wall fibrosis and occasional fibroblastic foci surrounded by honeycombing and normal alveoli were confirmed by post-mortem observation as thick-walled cystic lesions (TWCLs). Emphysematous destruction and enlargement of membranous and respiratory bronchioles with fibrosis were observed in the TWCLs. The cystic lesions were always larger than the cysts of honeycombing. The prevalence of both radiological and pathological TWCLs was 72.7% among CPFE patients, but no such lesions were observed in patients with IPF or emphysema alone (p = 0.001). The extent of emphysema in CPFE patients with TWCLs was greater than that in patients without such lesions. Honeycombing with emphysema was also observed in 11 CPFE patients.

**Conclusions:**

TWCLs were only observed in the CPFE patients. They were classified as lesions with coexistent fibrosing interstitial pneumonia and emphysema, and should be considered an important pathological and radiological feature of CPFE.

## Background

There have been several reports of idiopathic interstitial pneumonia, occasionally coexisting with emphysema [1,2], and a recent case series reported upper lobe emphysema associated with lower lobe fibrosis as a unique disorder termed ‘combined pulmonary fibrosis and emphysema (CPFE)’ [3]. Although Cottin *et al.* and Kitaguchi *et al.* reported case series’ of pathologically diagnosed CPFE with partial resection of the lungs performed via video-assisted thoracic surgery in 8 out of 61 [3] and 6 out of 47 [4] patients, respectively, the pathological features of these cases do not necessarily facilitate determination of the complete CPFE picture since the whole lung was not imaged. Combined emphysema and fibrotic lesions have been reported with airspace enlargement with fibrosis (AEF) [5], smoking-related interstitial fibrosis (SRIF) [6], and respiratory bronchiolitis-associated interstitial lung disease (RB-ILD) with fibrosis [7], but do not necessarily complicate fibrosing interstitial pneumonia and hence do not imply a poor prognosis; they are considered localised forms of fibrosis with emphysema [8].

Clinical evaluation to differentiate the characteristics of pulmonary fibrosis and emphysema is often difficult, and diagnosis of pulmonary fibrosis is important to evaluate the clinical course, treatment options, and the risk of acute exacerbation of interstitial pneumonia in patients with CPFE. However, no study has examined whole-lung pathological findings to investigate the association between pulmonary fibrosis and emphysema in patients with CPFE. The present study was conducted to evaluate the characteristics of pulmonary fibrosis with emphysema. To the best of our knowledge, this is the first report of correlations among clinical, radiological, and whole-lung pathological findings in an autopsy series of patients with CPFE, and of a comparison of these findings with those of patients with idiopathic pulmonary fibrosis (IPF) and emphysema alone.

## Methods

### Patient selection

Autopsy records of 1455 patients who attended the Department of Respiratory Medicine, Japanese Red Cross Medical Centre, Tokyo, Japan, between 1995 and 2011 were reviewed. A total of 186 patients were pathologically diagnosed with interstitial pneumonia, emphysema, or lung cancer; IPF and emphysema were diagnosed by a combination of clinical characteristics, chest computed tomography (CT) scans, and pathological features using available clinical records and radiological images [9,10]. Eight of the 186 patients were diagnosed with IPF alone, 17 with emphysema alone, and 22 with CPFE due to a combination of IPF and emphysema [3]. A total of 139 patients were excluded from the study because there were few available clinical records or radiological images, or IPF was not diagnosed: 11 diagnosed with usual interstitial pneumonia (UIP) and emphysema, 19 with IPF alone, 19 with emphysema alone, 11 with interstitial pneumonia other than pathological UIP, considered non-UIP, and emphysema, 32 with non-UIP alone, 25 with interstitial pneumonia associated with the presence of connective tissue disease or any other cause, and 22 with lung cancer alone.

### Assessment personnel

Clinical characteristics were reviewed by 3 respirologists, chest CT scans were evaluated by 2 radiologists, and pathological findings were assessed by 2 pathologists with a focus on interstitial pneumonia with emphysema.

### Clinical characteristics

Smoking history, pulmonary function test results, treatment regimen, cause of death, and lung cancer characteristics, including histological type, primary site, clinical stage, and treatment, were retrospectively reviewed by consulting clinical charts.

### CT scanning protocol

CT scans were obtained with various scanners including HiSpeed Advantage and HiSpeed FX/i scanners (GE Healthcare Milwaukee, Wis. USA) and Asteion 4-section and Aquilion 64-section multidetector scanners (Toshiba Medical Systems Corporation Tochigi, Japan). Scans were obtained with the patient in the supine position at full inspiration. High-resolution CT images were reconstructed with 1.5–2-mm collimation and 10–20-mm slice intervals, and were obtained from 15 patients with CPFE, 4 patients with IPF alone, and 3 patients with emphysema alone. Contiguous 5–10-mm collimation (conventional) scan was also obtained. Intravenous injections of contrast medium were administered to almost all patients with lung cancer. All images were viewed at window settings optimised for assessment of the lung parenchyma (width, 1200–1500 HU; level, -700 to -600 HU) and mediastinum (width, 300–400 HU; level 30–60 HU).

The CT images were reviewed by 2 radiologists and final decisions on the findings were reached by consensus. The observers assessed the presence, extent, and distribution of areas of reticulation, interlobular septal thickening, honeycombing, architectural distortion, bronchiectasis, cystic airspaces, emphysema, ground-glass attenuation, airspace consolidation, thickening of bronchovascular bundles, parenchymal nodules, lymph node enlargement, pleural thickening, and pleural effusion. The definitions of the radiologic findings were based on the Nomenclature of the Fleischener Society [10].

### Clinical and radiological analyses

Patients were diagnosed with CPFE if the following criteria [3] were met: (1) presence of emphysema on CT scan, defined as well-demarcated areas of decreased attenuation in comparison with contiguous normal lung and marginated by a very thin (<1 mm) or no wall, and/or multiple bullae (>1 cm) with upper zone predominance; (2) presence of a diffuse parenchymal lung disease with significant pulmonary fibrosis on CT scan, defined as reticular opacities with peripheral and basal predominance, honeycombing, architectural distortion and/or traction bronchiectasis or bronchiolectasis; and (3) focal ground-glass opacities and/or areas of alveolar condensation that could be associated but were not prominent.

The pattern of interstitial pneumonia on the CT scans was examined in accordance with IPF guidelines [9], and was categorised into 1 of 3 groups—UIP pattern; possible UIP pattern; and inconsistent with UIP pattern—because emphysema is not included in the exclusion criteria for UIP pattern in the IPF guidelines.

IPF was diagnosed based on the following guidelines [9]: exclusion of other causes of interstitial lung diseases; the presence of a UIP pattern on CT; and a specific combination of CT and pathological patterns. Exclusion criteria were as noted in the IPF guidelines [9]: micronodules; extensive ground-glass opacities; consolidation; or a peribronchovascular-predominant distribution leading to the consideration of an alternative diagnosis. Additionally, the presence of connective tissue disease or any other interstitial lung disease such as drug-induced interstitial lung disease, pneumoconiosis, hypersensitivity pneumonitis, sarcoidosis, pulmonary histiocytosis, lymphangioleiomyomatosis, and eosinophilic pneumonia was excluded.

Emphysema was defined as above on the basis of CT scans [3,10]. Centrilobular and paraseptal emphysema were graded into 3 categories based on the affected lung area: <25%, 25–50%, and >50% [11].

Thick-walled cystic lesions (TWCLs) were defined radiologically as cysts measuring at least 1 cm in diameter and delineated by a 1-mm-thick wall in an area of the lung where reticulation and/or honeycombing was evident on CT images [12-14].

Almost all patients underwent a final CT scan within 1 month of death, except for 4 emphysema patients for whom CT scans were performed in the year preceding death.

### Pathological analyses

All the lungs were infused with 10% buffered formalin through the main bronchi of both the lungs. After fixation for 7 days, the lungs were cut into 1.5-cm thick coronal or horizontal sections centred around the hilus. In addition, lesions of interstitial pneumonia, emphysema, and lung cancer were sampled according to chest CT findings (CT-guided sampling), and over 50 slides including such lesions as well as normal lung tissues were prepared in at least 20 cases. After formalin fixation, histological sections were stained with haematoxylin and eosin (HE) and an elastic Van Gieson (EVG) stain. Pathological features of all patients were assessed to determine whether there was any correlation with their CT scans. In accordance with established guidelines [9], UIP was identified pathologically based on evidence of marked fibrosis/architectural distortion with or without honeycombing in a predominantly subpleural/paraseptal distribution with patchy involvement of lung parenchyma affected by fibrosis and fibroblastic foci. Emphysema was defined as abnormal, permanent enlargement of the airspaces distal to the terminal bronchiole accompanied by destruction of their walls and absent or subtle and mild fibrosis [15]. We analysed centrilobular and paraseptal emphysema in each patient.

TWCLs were pathologically defined as cystic lesions at the level of membranous bronchiole with dense fibrous wall, destruction of respiratory bronchiole and alveoli, and occasional fibroblastic foci. These cystic lesions were often apposed to honeycomb lesion. We examined the location and frequency of these cystic lesions throughout the lobes in cases with CPFE, IPF, and chronic pulmonary emphysema.

### Statistical analyses

Differences between groups were compared using Student’s *t*-test and the Chi-squared test for numerical variables, Fisher’s exact test for categorical variables, and analysis of variance for continuous variables. Statistical analyses were performed using Microsoft Excel 2007 and SPSS 16.0 (SPSS, Chicago, IL, USA) software. All values were expressed as the mean ± standard deviation (SD), with 2-tailed p-values <0.05 considered statistically significant.

### Informed consent

This study was approved by the Internal Review Board of the Japanese Red Cross Medical Centre (#2-2), and informed consent was obtained from the families of all patients.

## Results

### Clinical characteristics

Patient characteristics are shown in Table 1. All patients had a history of heavy smoking. Patients in the CPFE group were all male, whereas the IPF-alone group included 1 female and 7 male patients, and the emphysema-alone group included 2 female and 15 male patients. No patient had a history of exposure to obvious occupational inhalants. Forced expiratory volume in 1 s/forced vital capacity (FEV_1_/FVC%) was significantly lower in the emphysema-alone group compared with the CPFE and IPF-alone groups. The percent predicted diffusing capacity of the lung for carbon monoxide (DLCO%) was significantly lower in the CPFE group compared with the IPF-alone and emphysema-alone groups. Fourteen (63.6%) patients in the CPFE group and 6 (75%) in the IPF-alone group were treated with corticosteroids for acute exacerbation of interstitial pneumonia and acute respiratory failure at the terminal stage. Six (27.2%) patients in the CPFE group and 5 (62.5%) in the IPF-alone group died from acute exacerbation of interstitial pneumonia; there was no significant between-group difference. The most common cause of death in the CPFE and emphysema groups was lung cancer (p = 0.007), but in the IPF group, it was acute exacerbation of interstitial pneumonia. Nineteen CPFE patients (86.4%) and 17 (100%) emphysema patients, but only 1 patient in the IPF-alone group, had lung cancer complications (p < 0.001) (Table 2). No significant differences in histological type, primary lesion, or lung cancer stage were noted among the groups. There was trend for CPFE patients to receive chemotherapy more frequently and irradiation less frequently than the emphysema-alone patients.

**Table 1:** Comparison of clinical characteristics among the CPFE, IPF-alone, and emphysema-alone groups

	**CPFE**	**IPF**	**Emphysema**	
	**(n = 22)**	**(n = 8)**	**(n = 17)**	**p-value**
**Age, years**				
*Median*	73.5	74	78	0.6949
*Range*	60–95	55–88	50–84	
**Gender, F/M**	0/22	1/7	2/15	0.244
**Smoking history, pack years**				
*Median*	64	43	75	0.6405
*Range*	20–200	30–120	15–150	
**Pulmonary function test**				
*VC*	2.52 ± 0.72	2.34 ± 0.86	2.85 ± 0.61	0.5175
*%VC*	83.1 ± 22.1	68.0 ± 27.7	87.0 ± 12.4	0.2902
*FEV1*	2.01 ± 0.19	1.60 ± 0.24	1.57 ± 0.22	0.281
*FEV1/FVC,%*	76.8 ± 3.31	81.8 ± 4.45	55.6 ± 4.06	0.0007
*DLCO*	6.30 ± 3.89	9.68 ± 3.65	15.45 ± 6.34	0.0149
*%DLCO*	36.6 ± 17.5	57.1 ± 27.4	102.5 ± 58.1	0.0153
*DLCO/VA*	1.96 ± 0.77	2.97 ± 0.26	3.06 ± 1.48	0.1428
*%DLCO/VA*	44.0 ± 16.8	69.1 ± 10.1	69.3 ± 30.6	0.0988
**Treatment for IP**				
*Corticosteroids*	14 (63.6%)	6 (75%)	-	0.452
*Immunosuppressive agent*	1 (4.5%)	0	-	0.733
*Long-term oxygen*	5 (22.7%)	1 (12.5%)	-	0.48
**Cause of death**				
*Lung cancer*	9 (40.9%)	1 (12.5%)	13 (81.3%)	0.007
*Acute exacerbation of IP*	6 (27.2%)	5 (62.5%)	-	0.091
*Infection*	4 (18.1%)	1 (12.5%)	1 (6.3%)	0.521
*Heart failure*	2 (9.1%)	0	2 (12.5%)	0.611
*Other causes*	1 (4.5%)	1 (12.5%)	1 (6.3%)	0.729

CPFE, combined pulmonary fibrosis and emphysema.

IPF, idiopathic pulmonary fibrosis; IP, interstitial pneumonia.

**Table 2:** Comparison of characteristics of lung cancer among the CPFE, IPF-alone, and emphysema-alone groups

	**CPFE**	**IPF**	**Emphysema**	
	**(n = 22)**	**(n = 8)**	**(n = 17)**	**p-value**
**Patients with lung cancer**	19 (86.4%)	1 (12.5%)	17 (100%)	<0.001
**Histology of lung cancer**				
*Adenocarcinoma*	8 (42.1%)	0	9 (52.9%)	0.523
*Squamouse cell carcinoma*	8 (42.1%)	0	4 (23.5%)	0.386
*Large cell carcinoma*	1 (5.2%)	0	0	0.615
*Small cell carcinoma*	4 (21.1%)	1 (12.5%)	4 (23.5%)	0.199
**Primary site**				
*Upper lobe*	9 (47.4%)	0	8 (47.0%)	0.646
*Lower lobe*	9 (47.4%)	1 (12.5%)	9 (52.9%)	0.581
**Clinical stage**				
*IA*	0	0	0	-
*IB*	2 (11.8%)	0	3 (20%)	0.76
*IIA*	0	0	2 (13.3%)	0.288
*IIB*	2 (11.8%)	0	0	0.367
*IIIA*	2 (11.8%)	1 (12.5%)	0	0.002
*IIIB*	2 (11.8%)	0	3 (20%)	0.76
*IV*	10 (58.8%)	0	7 (46.7%)	0.51
**Treatment for lung cancer**				
*Surgery*	1 (5.9%)	0	3 (18.8%)	0.46
*Chemotherapy*	13 (76.5%)	0	6 (37.5%)	0.081
*Radiation*	2 (11.8%)	0	7 (43.8%)	0.086
*Best supportive care only*	5 (29.4%)	1 (12.5%)	5 (31.3%)	0.291

Values are the mean ± standard deviation.

IPF, idiopathic pulmonary fibrosis.

### Clinicoradiological assessment

**Figure 1:**
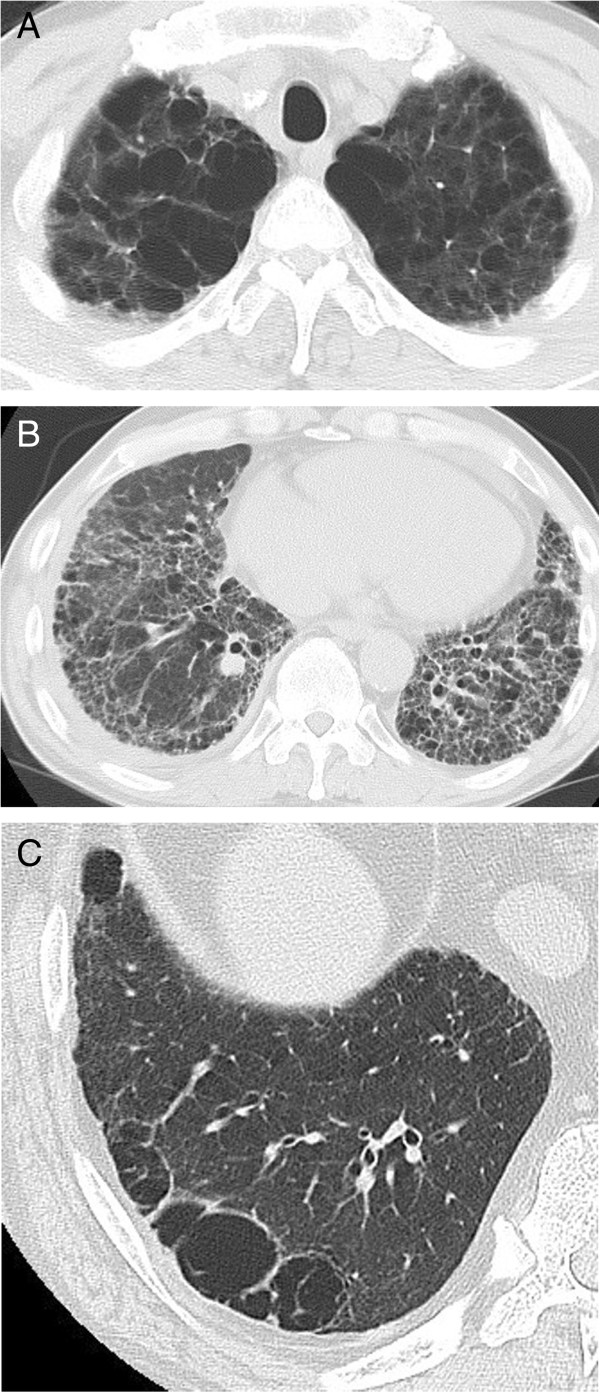
**Representative computed tomography (CT) scans performed within 1 month of death in a patient with combined pulmonary fibrosis and emphysema (CPFE) without lung cancer who died from pulmonary hypertension (A, B), and in another such patient with lung cancer who died from a non-respiratory cause (C). (A)** Upper lobe showing centrilobular and paraseptal emphysema and bullae. **(B)** Lower lobe showing reticular opacities with peripheral and basal predominance and honeycombing, which was diagnosed as definite usual interstitial pneumonia (UIP) pattern. **(C)** Lower lobe showing thick-walled cystic lesions (TWCLs) larger than honeycombing with peripheral reticular opacities.

**Figure 2:**
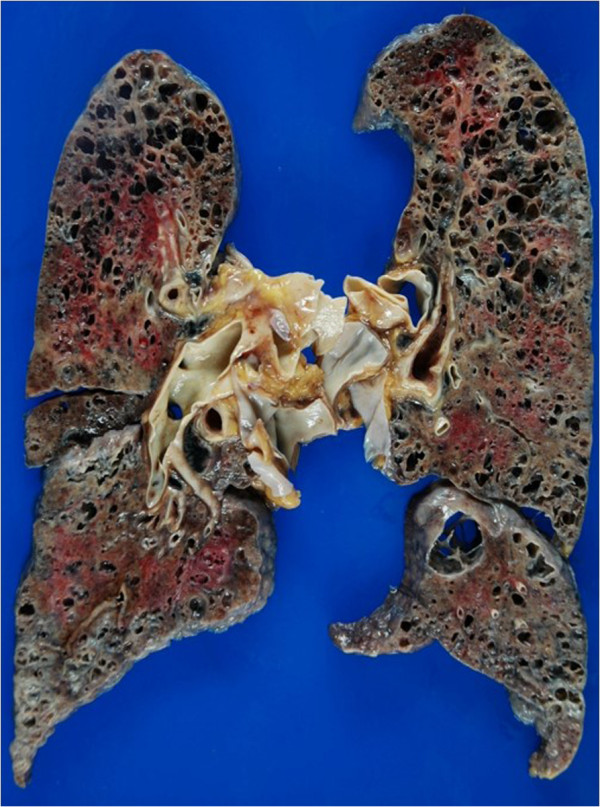
Gross photo showing marked upper lobe emphysema with fibrosis and lower lobe fibrosis and honeycomb lesion with emphysematous change in the lungs of a CPFE patient without lung cancer.

**Figure 3:**
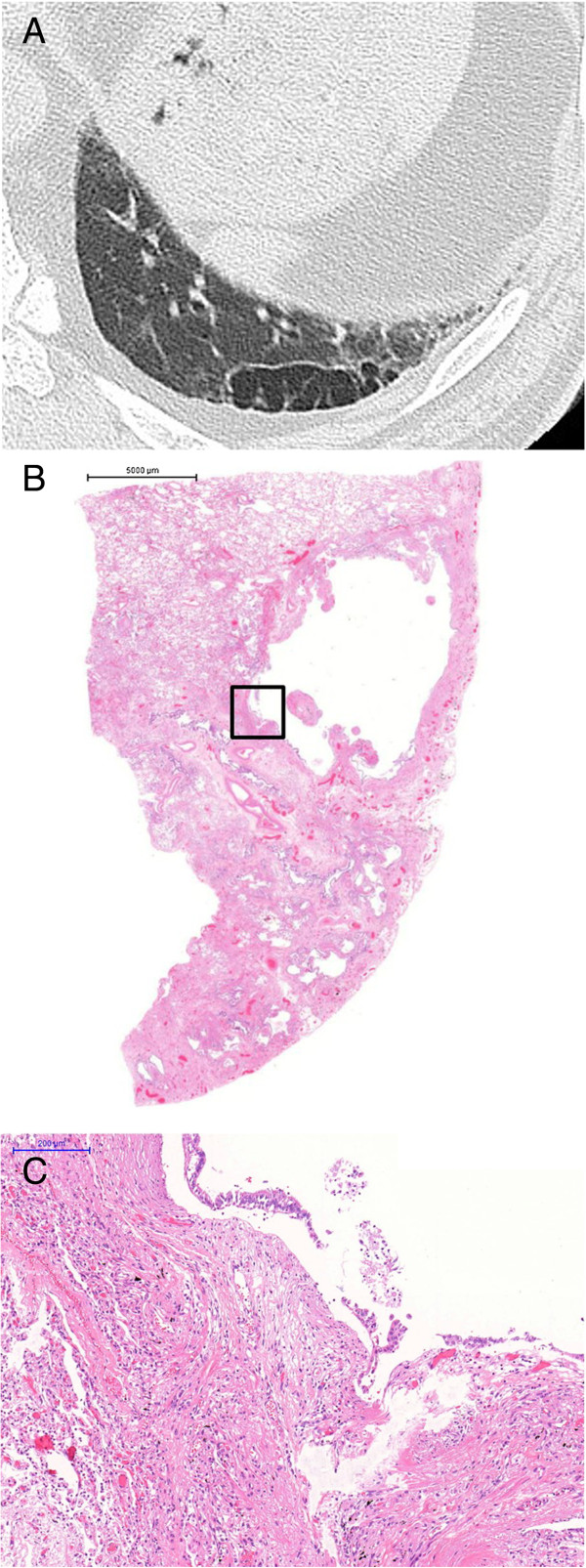
**Images of a CPFE patient with lung cancer in the right lower lobe who died from a non-respiratory cause. (A)** CT scan performed within 1 month of death; view of the left lower lobe showing TWCLs with slight reticulation on the pleural predominance. **(B)** Paramount view showing pathological TWCLs with fibrosis in the left lower lobe; corresponds to the CT scan (Figure 3-A). TWCLs involving the bronchiole and parenchyma with a dense fibrous wall beneath the terminal bronchioles. **(C)** High-power view of the square lesion in Figure 3-B showing fibroblastic focus in the fibrous walls.

**Figure 4:**
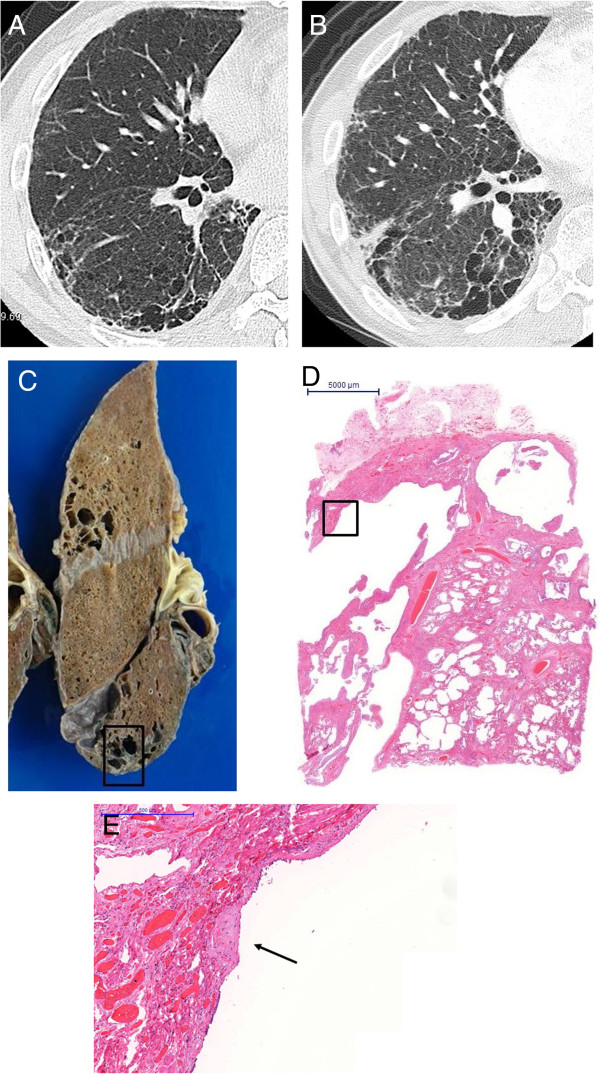
**Images of a CPFE patient without lung cancer who died from right heart failure owing to deterioration of pulmonary hypertension. (A)** CT scan performed 6 months prior to death; view of the right lower lobe showing TWCLs and traction bronchiectasis with reticulation on the pleural predominance. **(B)** CT scan performed within 1 month of death showing enlargement of TWCLs and simultaneous progression of reticulation despite smoking cessation. **(C)** Gross photo of the right lower lobe showing TWCLs (square). **(D)** Paramount view showing TWCLs apposed to honeycombing in the right lower lobe; corresponds to the CT scan (Figure 4-B) and gross photo (Figure 4-C). **(E)** High-power view of the square lesion in Figure 4-D showing fibroblastic focus (arrow) in the fibrous walls of the TWCLs.

**Figure 5:**
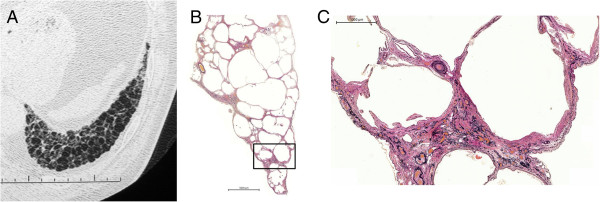
**Images of a CPFE patient with lung cancer who died from a pulmonary infarction. (A)** CT scan performed within 1 month of death showing honeycombing with a thin wall and the occasional integration of cysts in the left lower lobe. **(B)** Image of the lower lobe showing enlarged honeycomb cysts with thin walls and remnant of lung tissue therein corresponding to the CT scan (Figure 5-A). **(C)** High-power view of the square lesion in Figure 5-B showing perilobular atelectatic fibrosis with smooth muscle hyperplasia.

Representative images of emphysema in the upper lobe and diffuse parenchymal fibrosis in the lower lobe of the lungs of patients with CPFE are shown in Figures 1A-C and 2. Fifteen CPFE patients (68.2%) were diagnosed with a definite or possible UIP pattern and 7 (31.8%) with inconsistent with UIP patterns owing to evidence of peribronchovascular predominance and homogeneity; 8 IPF patients (100%) were diagnosed with definite UIP pattern (Table 3). TWCLs were observed in 16 (72.7%) CPFE patients: in the upper lobes of 68.8%, in the lower lobes of 62.5%, and in both lobes of 40% (Figure 3A). Enlargement of TWCLs was also observed with progression of reticulation in 5 of the 16 (31.3%) CPFE patients with TWCLs (Figure 4A-B). In contrast, TWCLs were not observed in any patients in the IPF- and emphysema-alone groups (p = 0.001). No patients with TWCLs had a history of recurrent pulmonary infection. The prevalence of honeycombing was 50% among the CPFE patients and 100% among the IPF patients. The prevalence of reticular opacity and consolidation was significantly higher among the IPF compared with CPFE patients. A representative image of the left lower lobe of the lung of a CPFE patient showing honeycombing with thin walls and the occasional integration of cysts is shown in Figure 5A. No significant differences in the type or degree of emphysema were noted between the groups.

**Table 3:** Comparison of radiological findings among the CPFE, IPF-alone, and emphysema-alone groups

	**CPFE**	**IPF**	**Emphysema**	
	**(n = 22)**	**(n = 8)**	**(n = 17)**	**p-value**
**TWCLs**	16 (72.7%)	0	0	0.001
*in upper lobe*	11 (68.8%)	-	-	-
*in lower lobe*	10 (62.5%)	-	-	-
**IP pattern**				
*UIP*	11 (50%)	8 (100%)	-	0.13
*Possible UIP*	4 (18.2%)	0	-	0.267
*Inconsistent UIP*	7 (31.8%)	0	-	0.084
**IP characteristics**				
*Honeycombing*	11 (50%)	8 (100%)	-	0.084
*Reticular opacity*	10 (45.5%)	8 (100%)	-	0.007
*Ground-glass opacity*	9 (40.9%)	2 (25%)	-	0.363
*Consolidation*	1 (4.5%)	3 (37.5%)	-	0.048
*Traction bronchiectasis*	1 (4.5%)	0	-	0.733
**Emphysema pattern**				
*Centrilobular*	15 (68.2%)	-	14 (82.4%)	0.265
*Paraseptal*	17 (77.3%)	-	9 (53.0%)	0.11
*Bullae*	11 (50%)	-	5 (29.4%)	0.195
**Degree of emphysema in upper lobe**				
*<25%*	4 (18.2%)	-	7 (41.2%)	0.111
*25–50%*	7 (31.8%)	-	5 (29.4%)	0.872
*>50%*	11 (50%)	-	5 (29.4%)	0.195

CPFE, combined pulmonary fibrosis and emphysema.

IPF, idiopathic pulmonary fibrosis; IP, interstitial pneumonia.

UIP, usual interstitial pneumonia; TWCLs, thick-walled cystic lesions.

### Pathological findings

Lung examination of the CPFE patients revealed emphysema with fibrosis in the upper lobes along with honeycombing and emphysema in the lower lobes (Figure 2). Additionally, in all CPFE and IPF-alone patients, diffuse parenchymal lung disease formed the UIP pattern and was accompanied by predominantly lower lobe contraction, honeycombing, and smooth muscle hyperplasia alternating with normal alveoli.

TWCLs were located in the centriacinar/centrilobular region, involving one or more acini, membranous and respiratory bronchioles with destruction of the alveoli and dense fibrosis of the walls along with occasional fibroblastic foci. TWCLs made no contribution to the diagnosis of UIP, however TWCLs were usually apposed to honeycomb lesion of UIP. TWCLs were observed in the lower lobes with adjacent normal parenchyma and honeycombing; importantly, they also continued to areas of honeycombing (Figures 3B-C, 4C-E). Furthermore, these cystic lesions were always larger than cysts of honeycomb lesions. On the other hand, TWCLs in the upper lobes were often observed with adjacent emphysematous parenchyma. The walls of the TWCLs were mainly composed of dense collagen; typically, mild inflammation with a patchy infiltrate of lymphocytes and plasma cells was present along with occasional fibroblastic foci. Areas with TWCLs were frequently lined by bronchiolar epithelium. Smooth muscle hyperplasia was seen in the interstitium as well as honeycombing. While the prevalence of pathological TWCLs was 72.7% among CPFE patients, TWCLs were not observed in any patients in the IPF- and emphysema-alone groups (Table 4).Furthermore, enlarged honeycomb cysts were observed in the subpleural area (Figure 5B) in 11 of the 22 (50%) CPFE patients, while such lesions were not observed in any patients with IPF-alone group. Pathologically, these enlarged cysts were 1 ~ 2 cm in size, which are larger than typical honeycombing and contained some remnant of lung tissue therein. The walls of enlarged honeycomb cysts are thin, but perilobular atelectatic fibrosis with smooth muscle hyperplasia were also observed in the periphery of these lesions (Figure 5C), as seen in typical honeycomb lesion of UIP.

**Table 4:** Comparison of pathological features among the CPFE, IPF-alone, and emphysema-alone groups

	**CPFE**	**IPF**	**Emphysema**	
	**(n = 22)**	**(n = 8)**	**(n = 17)**	**p-value**
**TWCLs**	16 (72.7%)	0	0	0.001
**IP pattern**				
*UIP*	22 (100%)	8 (100%)	-	-
**IP characteristics**				
*Honeycombing*	22 (100%)	8 (100%)	-	-
*FF*	6 (27.3%)	6 (75%)	-	0.027
*FF in TWCLs*	4 (25%)	-	-	-
**Other characteristics**				
*Bronchiolocentric fibrosis*	3 (13.6%)	2 (25%)	0	0.405
*DIP*	0	0	0	-
*RB*	3 (13.6%)	0	0	0.379
*DAD*	6 (27.3%)	6 (75%)	0	0.027

TWCLs, thick-walled cystic lesions; IP, interstitial pneumonia; UIP, usual interstitial pneumonia.

FF, fibroblastic foci; DIP, desquamative interstitial pneumonia; RB, respiratory bronchiolitis.

DAD, diffuse alveolar damage; CPFE, combined pulmonary fibrosis and emphysema; IPF, idiopathic pulmonary fibrosis.

The prevalence of fibroblastic foci was significantly higher among the IPF (75%) compared with CPFE patients (27.3%) (Table 4). Fibroblastic foci were observed in the TWCLs of 4 (25%) CPFE patients. Diffuse alveolar damage was significantly greater in the 6 patients with IPF without lung cancer (75%) compared with the 6 CPFE patients (27.3%), 5 of whom had lung cancer and experienced acute exacerbation after chemotherapy.

While no desquamative interstitial pneumonia (DIP) patterns were observed, respiratory bronchiolitis was observed in 3 CPFE patients (13.6%). Additionally, although we also observed bronchiolocentric fibrosis in 3 CPFE patients, this finding was neither diffuse nor prominent. Both centrilobular and paraseptal emphysema were observed, with centrilobular emphysema predominant in both groups. Neither diffuse fibrosis nor TWCLs were observed in the patients with emphysema alone.

### Evaluation of CPFE patients with TWCLs

The extent of emphysema in the CPFE patients with TWCLs was greater than that in the patients without TWCLs; however, other clinical findings were not significantly different among the groups (Table 5). Of the 6 CPFE patients who experienced acute exacerbation of interstitial pneumonia, 3 (50%) displayed TWCLs, and of the 15 CPFE patients with TWCLs, the 3 described above (20%) experienced acute exacerbation of interstitial pneumonia. Pathological assessment showed that the prevalence of diffuse alveolar damage was not significantly different between CPFE patients with and without TWCLs.

**Table 5:** Comparison of clinical, radiological, and pathological features among CPFE groups with or without thick-walled cystic lesions

	**CPFE**		
	**With TWCLs**	**Without TWCLs**	
	**(n = 15)**	**(n = 7)**	**p-value**
**Age, years**			
*Median*	73	68	0.7852
*Range*	60–86	64–95	
**Smoking history, pack years**			
*Median*	60	80	0.2954
*Range*	20–150	30–200	
**Extent of emphysema**			
*0-25%*	1 (6.7%)	3 (42.9%)	0.077
*25-50%*	4 (26.7%)	3 (42.9%)	0.387
*>50%*	10 (66.7%)	1 (14.3%)	0.032
**Pulmonary function test**			
*VC*	2.67 ± 0.63	2.05 ± 0.64	0.1672
*%VC*	87.37 ± 17.0	64.6 ± 23.7	0.0947
*FEV1*	2.09 ± 0.51	1.67 ± 0.32	0.1813
*FEV1/FVC,%*	74.6 ± 7.44	80.5 ± 11.2	0.3142
*DLCO*	6.93 ± 3.85	5.77 ± 4.56	0.733
*%DLCO*	39.5 ± 16.7	31.2 ± 21.5	0.5721
*DLCO/VA*	2.05 ± 0.8	2.48 ± 1.49	0.6024
*%DLCO/VA*	46.0 ± 17.4	56.3 ± 34.9	0.5778
**EsPAP**	51.5 ± 22.5	46.67 ± 9.07	0.7379
**Distribution of TWCLs**			
*Upper lobe*	12 (80%)	-	-
*Lower lobe*	11 (73.3%)	-	-
**Primary lesion of lung cancer**			
*Emphysema*	2 (14.3%)	0	0.455
*Fibrosis*	5 (35.7%)	3 (60%)	0.51
*TWCLs*	4 (26.7%)	-	*-*
*Others*	3 (20%)	2 (40%)	0.523
**Diffuse alveolar damage**	3 (20%)	3 (42.9%)	0.267

TWCLs, thick-walled cystic lesions.

CPFE, combined pulmonary fibrosis and emphysema.

EsPAP, estimated systolic pulmonary artery pressure.

## Discussion

This is the first report of correlations among clinical, radiological, and whole-lung pathological examinations in an autopsy series of CPFE. In the present study, we identified radiological and pathological TWCLs and described their association with CPFE. No pathological evidence of radiological TWCLs has yet been reported in the literature.

CPFE was described by Cottin *et al*. as a mainly radiological finding characterised by upper-lobe emphysema and lower-lobe fibrosis [3]; however, it is important to note that the present study showed a microscopic combination of fibrotic lesions with emphysema in the upper lobes and emphysematous lesions with honeycombing in the lower lobes.

The combination of fibrotic lesions and emphysema has been classified into 2 groups: diffuse forms of fibrosis with emphysema, and localised forms of fibrosis with emphysema [8]. AEF [5], SRIF [6], and RB-ILD with fibrosis [7] are categorised as localised forms, and Katzenstein *et al*. and Yousem suggested that SRIF and RB-ILD with fibrosis are not a diffuse fibrosing interstitial pneumonia. Kawabata *et al*. reported that AEF could be included as part of the spectrum of SR-ILD [5]; however, no correlation with radiological features has been reported thus far.

TWCLs are closely associated with smoking given the emphysematous destruction of bronchioles and parenchyma, and the fibrosis that occurred at the level of the membranous bronchiole and also respiratory bronchioles observed in the present pathological analysis. Although localized forms of fibrosis with emphysema do not appear to be part of diffuse fibrosing interstitial pneumonia, TWCLs are considered to be associated with smoking-related fibrosing interstitial pneumonia by pathological analysis because TWCLs include fibrosis and emphysema at the level of membranous and respiratory bronchioles, and are surrounded by the pathological diffuse parenchymal fibrosis with adjacent honeycombing, and sometimes continue to areas of honeycombing. TWCLs are also characterised by the combination of pulmonary fibrosis and emphysema because, radiologically, the extent of emphysema was greater in CPFE patients with TWCLs compared with those without such lesions. Moreover, TWCLs were not observed in patients with IPF or emphysema alone by both radiological and pathological analysis.

It has been reported that a history of smoking is present in all CPFE patients, suggesting that it may be a risk factor for CPFE [3], and smoking is also the major risk factor for chronic obstructive pulmonary disease [16]. In the present study, the median extent of smoking was 64 pack-years among the CPFE patients, which is high relative to the 5–73 pack-years reported in previous studies [17]. There was no significant difference in the smoking index between CPFE patients with and without TWCLs. However, the extent of emphysema among the CPFE patients with TWCLs was greater than that among patients without TWCLs. Emphysema was included in the destruction of bronchioles and parenchyma and the fibrosis at the level of membranous and respiratory bronchioles of the pathological TWCLs, which may explain the relationship between TWCLs and smoking. In addition, it has been reported that only about 15–25% of smokers develop airflow obstruction, and there is a substantial component of genetic susceptibility associated with the development of COPD [18]. Smoking and genetic polymorphisms have also been identified as potential risk factors for the development of IPF [19]; therefore, we speculate that there may be differences in individual susceptibility to the development of TWCLs or typical CPFE in a unique subset of patients exposed to smoking.

TWCLs were differentiated from AEF [5], SR-IF [6], and RB-ILD with fibrosis [7] by the presence of fibroblastic foci, their association with fibrosing interstitial pneumonia and honeycombing, and their size (Table 6). In the present study, TWCLs containing fibroblastic foci were confirmed by post-mortem observation of radiologically identified TWCLs. Thus, TWCLs are considered an important radiological/pathological feature of CPFE. TWCLs are also considered combined lesions of active parenchymal fibrosis and emphysema and should, therefore, be managed not only as emphysema, but also as fibrosing interstitial pneumonia.

**Table 6:** Histological comparison between TWCLs, localized forms of fibrosis with emphysema, and honeycombing of UIP

	**TWCLs**	**Localized forms of fibrosis with emphysema (8)**	**Honeycombing**
Origin	membranous bronchiole	no definition	peripheral alveoli
Size	>10 mm	no definition	3 ~ 10 mm
Association with emphysema	(+)	(+)	(-)
Fibroblastic foci	(+)	(-)~(+)	(+)
Association with fibrosing IP	(+)	(-)~(±)	(+)

IP, interstitial pneumonia; TWCLs, thick-walled cystic lesions.

Radiological features of CPFE include large, relatively thick-walled cysts in addition to pulmonary emphysema, and some large cysts may grow within areas with interstitial pneumonia [14]. In the present study, radiologically, gradual enlarging of TWCLs simultaneous with gradual progression of fibrosing interstitial pneumonia was observed despite smoking cessation in 5 CPFE patients with TWCLs. This suggests that enlargement of TWCLs is probably indicative of deterioration of fibrosing interstitial pneumonia, and in the present study, these progressive changes could be observed in parallel.

Occasional fibroblastic foci are present in diffuse forms of fibrosis and emphysema [8]. Kawabata *et al*. reported that the incidence of acute respiratory failure was 0% in patients with AEF without a UIP pattern, maybe because AEF is defined without fibroblastic foci [5]. On the other hand, Katzenstein *et al*. reported that fibroblastic foci are included in SRIF [6], but this lesion is not a diffuse interstitial pneumonia. In the thick-walled honeycomb of IPF patients, there are significantly higher percentages of diffuse alveolar damage compared with the thin-walled type [20]. In the present study, all CPFE patients with TWCLs were diagnosed with diffuse forms of fibrosis with emphysema, and a pathological UIP pattern was observed in all patients; therefore, acute exacerbation of interstitial pneumonia should be considered a risk in CPFE patients with TWCLs. However, the prevalence of acute exacerbation could be lower in CPFE compared with IPF patients, as is indicated by this study, because the prevalence of fibroblastic foci and diffuse alveolar damage was significantly higher in patients with IPF alone. Further research to determine whether acute exacerbation is more frequent in patients with IPF alone compared with CPFE is needed because of the small number of patients included in this study.

Honeycombing with emphysematous destruction was observed in half of the CPFE patients, with enlargement and sometimes thinning of the walls, which also composes the UIP pattern, indicating a pathological characteristic of CPFE. Honeycombing with thin walls and enlargement have previously been observed in CPFE patients [1], and we also noted these findings in our patients. However, these features were not present in patients who had IPF alone, indicating that emphysema is the likely cause of the enlargement and thin walls. Such honeycombing is implicated as a pathological expression of honeycombing combined with emphysema. According to pathological analysis in the present study, honeycombing with emphysema is essentially different from TWCLs because the former is absolutely honeycombing in a perilobular distribution with fibrosis of lung parenchyma. On the other hand, TWCLs make no contribution to the diagnosis of UIP because, in addition to essential difference from honeycombing, the membranous bronchiole is implicated as an origin of TWCLs, and TWCLs involve respiratory and membranous bronchiole lesions in centriacinar/centrilobular distribution. However, TWCLs are lesions representative of a combination of fibrosing interstitial pneumonia and emphysema, and it is importantly demonstrated that TWCLs coexist with honeycombing and UIP, and TWCLs sometimes continue to areas of honeycombing.

At HRCT TWCLs represent enlarged cysts with thick walls even in an area of the lung where honeycombing is not evident, and honeycombing with emphysema also represent enlarged cysts with sometimes thin walls only in an area of honeycombing. Therefore, thickness of the walls and absence of pre-existing honeycombing could be differential point between these lesions. However, radiologically, honeycombing mixed with TWCLs may mimic honeycombing consisting of enlarged cysts.

Respiratory bronchiolitis is extremely common in smokers, and was present in 100% of young smokers examined by Niewoehner *et al.*[21] in their landmark paper, and in 100% of current smokers reviewed by Fraig *et al*. [22]. Our case series had a very small number of patients with RB and no patients with RB-ILD; therefore, there is a discrepancy in the proportion of patients in whom RB/RB-ILD was identified. Some of these differences might be explained by formalin inflation of the lungs and the “washing out” of the pigmented macrophages [5].

Lung cancer develops more frequently in patients with IPF and COPD; the reported incidence of lung cancer is 22.4–31.3% in IPF patients [23,24] and 6.8–10.8% in COPD patients [25]. In the present study, patients who died from lung cancer diagnosed at autopsy were included in the 186 patients because lung cancer was considered a complication of interstitial pneumonia or emphysema. Although there is selection bias due to autopsy series, the lower number of patients with lung cancer in the IPF-alone group compared with the CPFE and emphysema-alone groups might be related to the high prevalence of diffuse alveolar damage among patients in the IPF-alone group. In this context, the fact that the number of patients with lung cancer was significantly lower in the IPF-alone group compared with the CPFE and emphysema-alone groups suggests that emphysema could more frequently complicate lung cancer than pulmonary fibrosis. On the other hand, lung cancer tended to develop close to areas of dense fibrosis with architectural distortion in the CPFE patients, particularly in the 8 (42.1%) patients with fibrotic lesions and the 4 (21%) patients with TWCLs. Thus, TWCLs, which are lesions that combine fibrosing interstitial pneumonia and emphysema, could also be considered a source of lung cancer development.

Several limitations to the present study warrant mention. First, the present study was subject to selection bias, because only autopsy series patients who provided consent for such a study or whose family provided such consent were evaluated. Second, 14 CPFE patients (77.8%) were treated with corticosteroids, which could potentially influence the pathological findings in terms of the patterns of pulmonary fibrosis and the number of fibroblastic foci. Third, pulmonary hypertension (PH), one of the most severe complications of CPFE, was not examined, because right heart catheterisation could only be assessed for a PH diagnosis in one patient due to the need for an invasive investigation. Right heart catheterisation remains the ‘gold standard’ for the diagnosis of PH, but in clinical practice non-invasive investigations are performed for most patients. Fourth, a pathological UIP pattern of interstitial pneumonia was observed in all patients in the CPFE and IPF-alone groups in the present study. Patients with non-UIP patterns with or without emphysema and secondary interstitial pneumonia were excluded, and so patients with pathological UIP and non-UIP or secondary interstitial pneumonia could not be compared.

CPFE illustrates the limitations of a simplistic diagnostic dichotomy between lung emphysema and fibrosis because CPFE is diagnosed with radiological criteria. Although TWCLs may be misunderstood as lesions of solely emphysematous changes radiologically, they were only observed in CPFE patients and were considered combined lesions of active parenchymal fibrosis and emphysema by pathological evaluation. Thus, the evaluation of TWCLs contributes to the diagnosis, treatment, complication, prognosis, and the risk of acute exacerbation of interstitial pneumonia in CPFE patients.

## Conclusions

In the present autopsy series, TWCLs were only observed in the CPFE patients. TWCLs are considered to be associated with fibrosing interstitial pneumonia and emphysema, and should be considered an important radiological and pathological feature of CPFE.

## Competing interest

None of the authors have any financial or personal relationships with other individuals or organisations that could inappropriately influence the work reflected in this manuscript.

## Authors’ contributions

MI and SI: Participated in the design of the study and the collection, analysis, and interpretation of data; performed the statistical analysis; drafted the manuscript. YK: Participated in the collection, analysis, and interpretation of data. NY and FS: Participated in the interpretation of the radiography images of the autopsy series. TK: Participated in interpretation of the pathology findings for the autopsy series. TO, AA and AG: Participated in the design of the study and the interpretation of data. TT: Participated in the design of the study, the collection and analysis of data, the interpretation of the pathology findings from the autopsy series. All authors critically reviewed the manuscript in relation to important intellectual content. All authors read and approved the final manuscript.

## Pre-publication history

The pre-publication history for this paper can be accessed here:

http://www.biomedcentral.com/1471-2466/14/104/prepub
